# Accurate Permittivity Measurements for Microwave Imaging via Ultra-Wideband Removal of Spurious Reflectors

**DOI:** 10.3390/s100908491

**Published:** 2010-09-10

**Authors:** Mathew G. Pelletier, Joseph A. Viera, John Wanjura, Greg Holt

**Affiliations:** 1 United States Department of Agriculture, Agricultural Research Services, Lubbock TX, 79403 USA; E-Mails: John.Wanjura@ars.usda.gov (J.W.); Greg.Holt@ars.usda.gov (G.H.); 2 Sensors Group Microsemi Corp. Lowell MA, 01851 USA; E-Mail: jviera@microsemi.com

**Keywords:** ultra-wideband, uwb, microwave imaging, permittivity, moisture sensing, hidden object detection

## Abstract

The use of microwave imaging is becoming more prevalent for detection of interior hidden defects in manufactured and packaged materials. In applications for detection of hidden moisture, microwave tomography can be used to image the material and then perform an inverse calculation to derive an estimate of the variability of the hidden material, such internal moisture, thereby alerting personnel to damaging levels of the hidden moisture before material degradation occurs. One impediment to this type of imaging occurs with nearby objects create strong reflections that create destructive and constructive interference, at the receiver, as the material is conveyed past the imaging antenna array. In an effort to remove the influence of the reflectors, such as metal bale ties, research was conducted to develop an algorithm for removal of the influence of the local proximity reflectors from the microwave images. This research effort produced a technique, based upon the use of ultra-wideband signals, for the removal of spurious reflections created by local proximity reflectors. This improvement enables accurate microwave measurements of moisture in such products as cotton bales, as well as other physical properties such as density or material composition. The proposed algorithm was shown to reduce errors by a 4:1 ratio and is an enabling technology for imaging applications in the presence of metal bale ties.

## 1. Introduction

The use of microwave imaging is becoming more prevalent for detection of interior hidden defects in manufactured and packaged materials. Applications range from detection of hidden weapons at airports to detection of dangerously high moisture areas in packaged and baled fiber products such as raw cotton lint. In applications for detection of hidden moisture, microwave tomography can be used to image a bale and then perform an inverse calculation [1–3] to derive an estimate of the variability of the hidden interior moisture, thereby alerting personnel to damaging levels of unseen moisture [4,5] before fiber degradation occurs. One impediment to this type of imaging is when the packaging utilizes metal strapping ties, which create large deviations in measured signal propagation. Thus, to date, it has not been possible to perform accurate microwave measurements of moisture on bales that are tied with metal strapping. As currently 40–50% of the market utilizes metal strapping in their bale packaging systems, a solution to allow for imaging of internal bale moisture is highly desirable by the industry.

Scattering of electromagnetic radiation from a sphere or long cylinder in which the sphere or infinitely long cylinder are significantly smaller than the wavelength, results in a scattering, quantified by classification as Rayleigh scattering, or as noted in radar handbooks, long thin wires that exhibit Mie scattering even though the diameter of the wire would normally place the scattering in the Rayleigh region [6–8]. Thus, while the size of the reflector has a significantly reduced radar cross-section, due to the long wire that radiates, it still reflects a significant amount of energy even with the reduced cross-section. This paper compares the results of predicted Mie scattering, via simulations of Maxwell’s electromagnetic equations, to experimental results obtained with microwave imaging antennas. Of primary interest is a typical sensing configuration with close antenna proximity to the metal bale ties. It is hypothesized that the scattering is causing destructive and constructive interference as the bale ties are conveyed past the imaging antenna array and thereby are transported into and out of the antenna’s sensing region. The primary objective of this research was to develop methods by which to mitigate the influence of metal ties on the electrical permittivity measurements, thereby providing a means for producing microwave images of internal moisture content that are clean from interference from the close proximity of metallic strapping at high density, 15–20 cm, spacing.

## 2. Experimental Section

**Figure 1.**
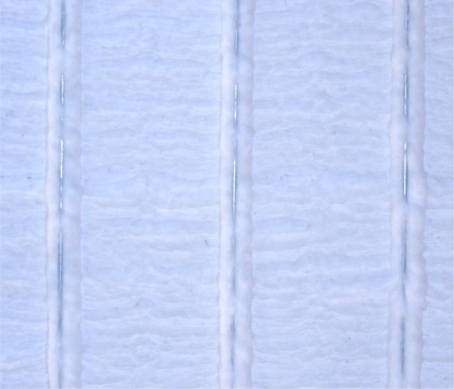
Close up view of the metal wire-tires on a commercial cotton bale. For reference the ties are spaced at 17 cm between ties.

**Figure 2.**
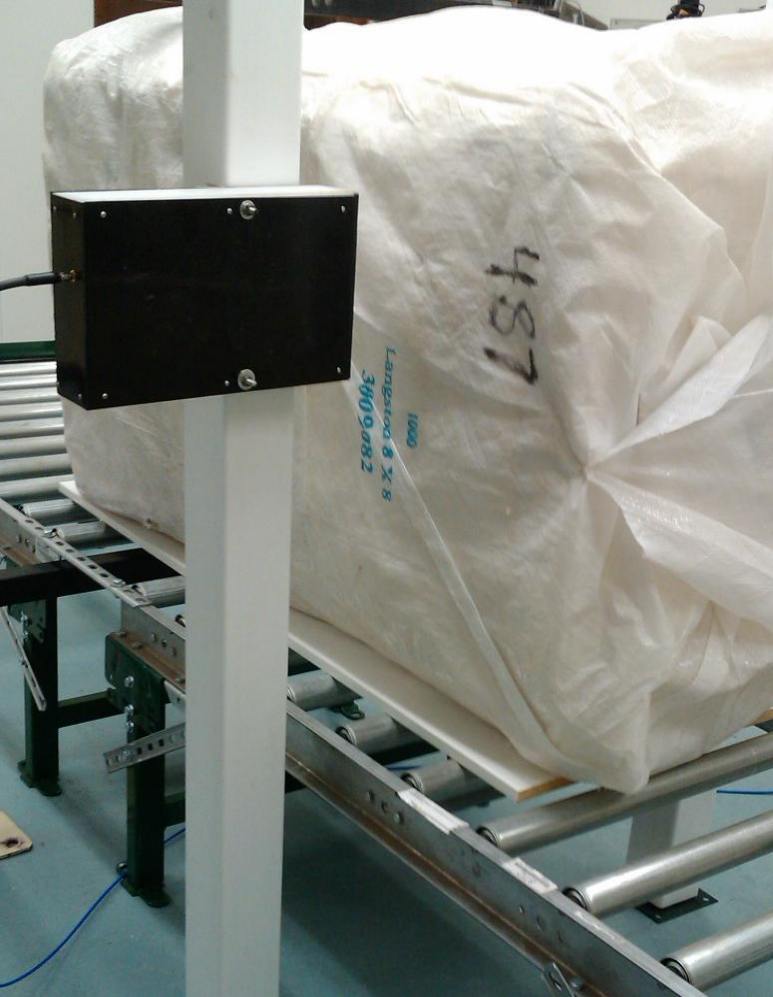
Experimental apparatus to convey the cotton bales past the imaging antennas to allow measurement of the signal response at specific targeted discrete positions along a bale.

**Figure 3.**
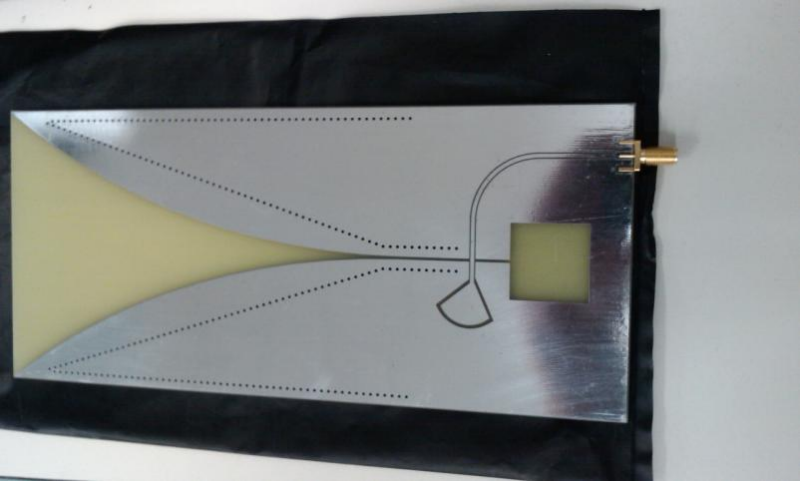
Wideband planar fan beam antenna.

To quantify the influence of the metallic ties, used in packaging of the bales, Figure 1, on the measured signal, a uniform density, “UD” cotton bale, measuring 53 × 84 × 137 cm, was tied with eight industry standard steel strapping ties, with a 3 × 4 mm cross sectional diameter wire located at a spacing of 17 cm. The experiment utilized a bale that was conditioned over the course of several months to ensure a uniform internal equilibrium moisture content of 5.6%, (wet-basis; per cotton industry standard reporting). The moisture content was determined per standard protocols via convection oven based gravimetric analysis [9,10]. At the time of the test, the bale weight was measured to be 220 kg, a typical production weight for commercial bales. The use of a uniformly conditioned bale limited the variation in the microwave signal response to the near proximity of metal bale ties to the scanning antennas as the location of the scan was varied as the bale was conveyed past the antennas. Also of interest is that this particular bale had a slight density taper to it which provided a linear slope to the measured permittivity as the scan progresses from one end of the bale to the other. A single set of scanning antennas were setup on either side of a conveyor for a through beam free-space measurement, Figure 2. The signal response was obtained across the frequency range from 500 MHz to 2.5 GHz utilizing a wide-band planar fan beam antenna, Figure 3. The antennas were setup in an off-center-axis, as shown in Figure 2, so-as to match the typical configuration utilized for industrial deployment due to the need to make room for the conveyance chain trolleys utilized in the typical automatic bale bagging-weighing systems.

The testing consisted of placing the bale at a static position in front of the conveyor and taking a frequency sweep to obtain scattering parameters in the S21 direction, as provided by an HP 8753D network analyzer utilizing an in-place air-path through calibration. The air-path calibration was obtained without the bale in the field of view and was utilized as a reference to remove the influence of the cabling, antennas and internal instrument variation and path lengths. After the air-reference was taken, the bale was then transported into position and a frequency scan was obtained for each position. After each scan the bale was indexed forward by 1 cm and the process was repeated. Thus, the testing obtained a frequency scan for each position as the bale was indexed past the antennas. The choice of the reported frequencies were chosen from the available frequencies that were determined to have an optimal standing wave ratio, “SWR”, match [11] with the criteria for the SWR to be lower than 1.6, so as to provide the most ideal measurements.

**Figure 4.**
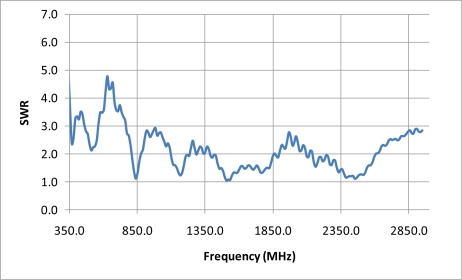
SWR response of the imaging antenna, of Figure 1, across the frequency range from 350 MHz through 2.95 GHz.

The second phase of the experiment repeated the bale scan with the replacement of the network analyzer with an ultra-wideband system configured with a 60 ps rise-time impulse generator and 12 GHz bandwidth sampling signal-correlator, such as is typical in ultra wide-band systems [12]. The impulse signal was then subsequently band-limited by the antennas, to an effective bandwidth from 700 MHz to 2.95 GHz, as detailed in the SWR plot of Figure 4.

For additional supporting evidence for this research, a finite-difference-time-domain “FDTD” electromagnetic simulation model, that was based on Yee’s seminal paper [13] and extended by the authors [13] to include internal variability in complex permittivity, was utilized. The FDTD model was used to explore the influence of the metal bale ties, on the signal quality, as a function of the relative position to the receiving antenna and the interrogation frequencies. The model simulated the metal bale-ties as a cylindrical metal scatterer with the computational boundary terminated with a perfectly-matched absorbing boundary layer, “PML”, executed as a split-field Berenger PML. The simulation utilized a fixed frequency sinusoidal oscillator at frequencies ranging from 1.0 GHz to 5.0 GHz. The metallic bale-ties were embedded directly into the model as normal nodes, with the conductivity term set to the appropriate values for steel. The node spacing was set at 20–50 nodes per wavelength, depending upon the frequency, with the time step set to two times delta-x divided by the speed-of-light for model stability, per the normal Courant stability model, on an orthogonal Cartesian coordinate system [14–17].

## 3. Results and Discussion

**Figure 5.**
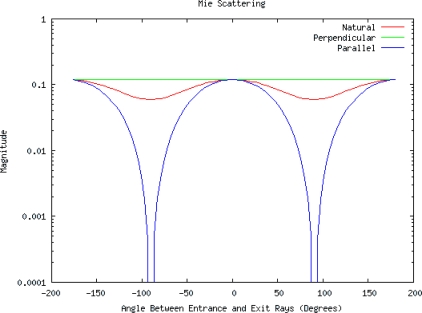
Mie scattering prediction curves for ratios {0.008, 0.014, 0.020} (cylinder-diameter to wavelength ratio) for the frequencies {914, 1720, 2450 MHz} respectively. We note here the natural curve in red is un-polarized radiation. Also of interest is the imaging antennas are in parallel polarization to the infinite cylinder model (metal bale ties).

In examination of Figure 5, Mie scattering theory [6–8] suggests antenna to wave-front angles as high as 50–70 degrees can still push significant multipath energy onto the receiving antenna. Further, as in the normal course of utilization in a production environment, the bales don’t always track exactly along the same path. Therefore one of the key design criteria’s is a system that is immune to limited side to side variability which in turn translates into variations in distance between the relative distance from the antenna to the bale’s edge where the ties are located. Mie scattering theory [6–8], coupled with the bale location variability, would likely preclude the use of a simple inverse channel equalizer-filter such as a Wiener-Hopf [18] equation to filter out the influence of the channel variations created by the multipath rich environment due to the metal bale ties as the bale to antenna geometry will likely vary from one bale to the next, thereby altering the required channel equalizer that the Wiener-Hopf equation would provide. While it is possible that an adaptive filter [18] could be used, the lack of a suitable noise input sensor would likely also preclude its use.

In working towards an understanding of the influence of the metal bale ties on the system, utilizing a first-order ray-tracing analysis of a multi-path signal propagation model [13], provides analytical support to the experimental evidence that the wavelength dependence of the constructive and destructive interference, is a result of the combination of the wavelength and distance from the scattering metallic tie to the antenna in relation to the direct path length; *i.e.*, the combination of the scattered wave to the direct path wave is a function of wavelength and proximity spacing. Thus, this model supports the experimental results which shows fluctuations in the peak relative location in the measured phase delay, even thought the bale is of nominally uniform moisture and density across the entire volume of the bale.

**Figure 6.**
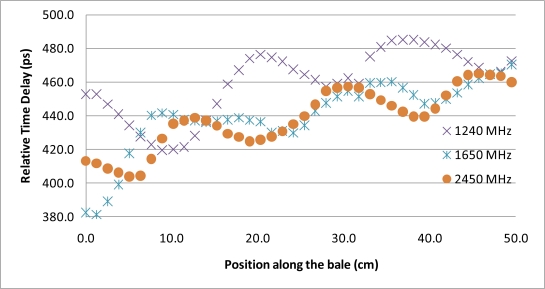
Response of an imaging antenna pair under the influence by metal bale ties, showing the metal bale-tie created varying phase delay response, as the bale is conveyed past the sensor along the x axis in the figure below. Of interest is the distances between the peaks in the phase delay shown are nearly the same distance as the metallic wire-tire spacing’s on the bale. Also of note is that the tested bale was configured with uniform moisture content with a slow linear-taper in density, thus the correct response would be a linear line with a positive slope (4:5) from right to left, free from oscillations.

Of particular note is in the comparison of the Mie scattering magnitude response of Figure 5, to the received phase response from experimental results, Figure 6, which suggests a varying degree of destructive and constructive interference between the direct path and the scattered-path is producing a phase shift that is a function of frequency as well as the relative positioning between the metal bale ties and the antennas.

**Figure 7.**
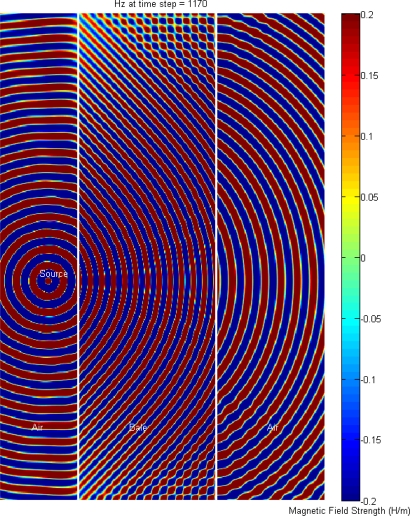
FDTD simulation result illustrating the advancing wave-fronts, as they propagate from left to right, in a bale without metal ties.

**Figure 8.**
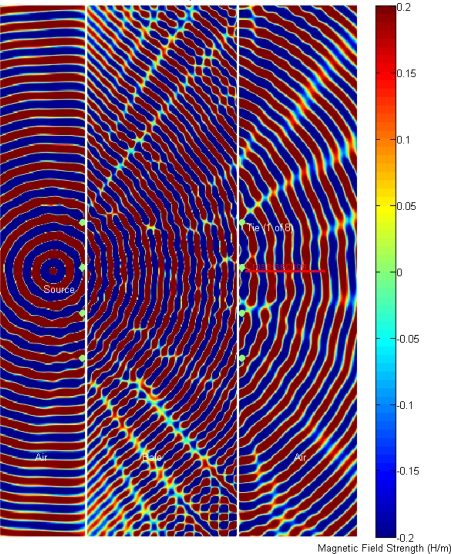
FDTD simulation prediction showing constructive-destructive interference as the advancing wave-fronts propagates from left to right, magnetic field strength, “H/m”, with the top view showing the 2.5 GHz simulation and the 2.0 GHz simulation in the bottom view. Of particular interest are the ripples or delays in the advancing front that are to the right and downstream from the metal bale-ties where the antennas would be located which would translate to an additional delay in the measured phase due to the ripples created by the Mie scattering.

To gain further insight into how the metallic bale-ties are influencing the signal propagation and hence the measured electrical permittivity, an FDTD simulation was conducted to compare the propagation for the case of the bale without metallic ties, Figure 7, to the case where the bale has metal wire-ties, Figure 8. The simulation predicts that transmit-side wire-ties also contribute to the signal degradation, in addition to the expected receive-side wire-ties.

**Figure 9.**
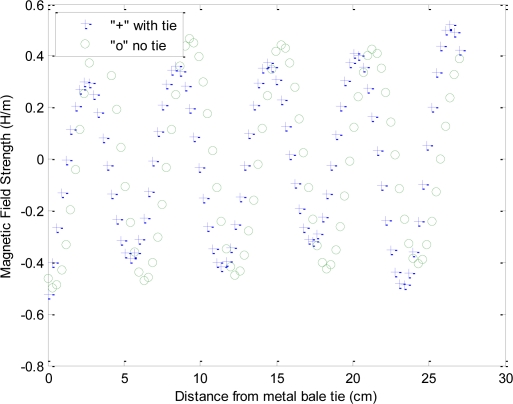
Comparison of the two test cases, with *versus* without wire-ties from the final steady-state FDTD simulation, illustrates both the magnitude and phase shift that occurs due to the wire-ties.

To gain further insight into the impact of the two test FDTD cases, with *versus* without wire-ties, the cross-sectional area, depicted in red “cross-section” in Figure 8, was captured at the final steady-state time step and plotted for comparison. The results indicate the wire ties are expected to contribute a significant shift in the phase delay as well as an amplitude shift (Figure 9).

**Figure 10.**
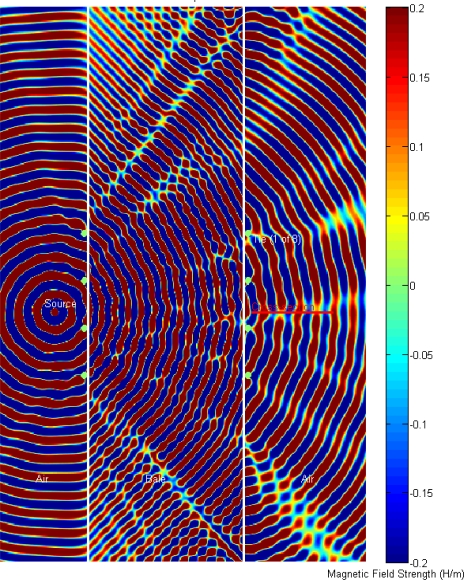
Another comparison of the two test cases, with *versus* without wire-ties from the final steady-state FDTD simulation, for a different offset from the wire-ties, again illustrating both the magnitude and phase shift that occurs due to the wire-ties. Lower view shows the cross-section comparison between the two test-cases.

**Figure 11.**
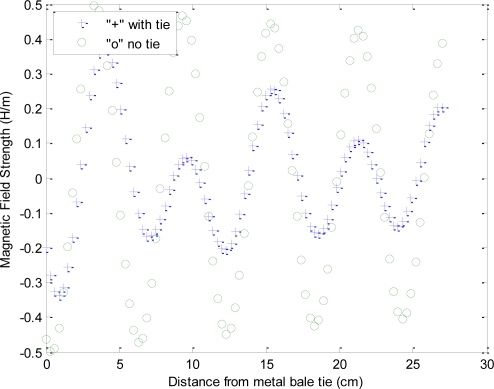
Another comparison of the two test cases, with *versus* without wire-ties from the final steady-state FDTD simulation, illustrates both the magnitude and phase shift that occurs due to the wire-ties.

Figure 10 is a repeat of the experiment with a shift in the relation between the transmit antenna and the wire-ties with impact on the phase detailed in Figure 11.

**Figure 12.**
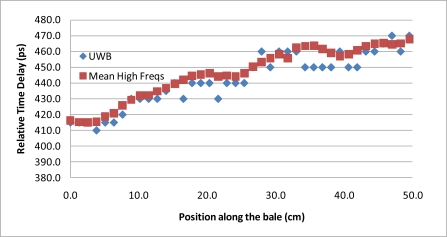
Results detail of the proposed techniques showing the effective removal of the noise created by the metal-ties. The first technique utilized an ultra wideband imaging antenna excited with a 60 ps impulse (antenna bandwidth range: 700–2,600 MHz). The second technique depicted is an artificial ultra wideband response constructed analytically from a stepped-frequency scan obtained via a network analyzer. Note each data point in the x direction corresponds to a specific bale location.

**Figure 13.**
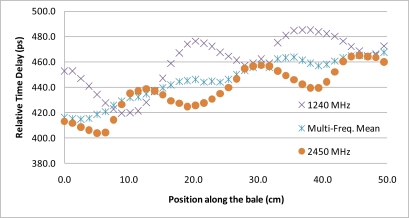
Comparison to single frequency estimate of propagation delay to an estimate derived from multiple frequencies showing a 4:1 reduction in signal ripple.

The second phase of the experiment was a repeat of the bale scan experiment utilizing ultra-wideband, “UWB”, signals and time-domain processing. The original justification for the addition of the UWB testing to the protocol was to verify that the assumed phase delay integer-n roll-over, utilized for resolving the phase ambiguity for the network analyzer measurements, was correct. The testing however yielded the surprising results of near complete suppression of the metal bale ties from the received signal. A repeat of the experiment utilizing the same locations and correlated to the network analyzer scans provides strong support that the use of UWB signals can be used to remove the impact of the Mie scattering caused by the wire-ties. Qualitatively, in Figure 6, it can be seen that each frequency is peaking at a different offset from the wire-tie. Thus, the combination of the frequencies would logically combine to offset each other, similar to the mathematical convergence that occurs in an infinite-series. To further test the concept of utilization of the full spectrum to remove the effects of the wire-tie scattering, the UWB time delay measurements were compared to an analytical composite that joins frequencies, via arithmetic mean, from across the range from 1 GHz to 2.45 GHz, Figure 12. The results suggest that by spreading the signal out across the complete spectrum and letting the received signal be comprised of the composite of all the frequencies, the destructive and constructive interference from the metal bale ties effectively cancel each other out, yielding a clean measurement substantially free from the Mie scattering artifacts. The magnitude of the improvement can be seen as detailed in Figure 13 where the improvement provided by utilization of multiple frequencies, in comparison to a single frequency estimate of propagation delay, is a 4:1 reduction in signal ripple. This is significant as it allows for imaging applications that utilize spot estimates of the permittivity of the material as it provides significant improvements to be gained at each location that are substantially free from bale tie interference thereby allowing for the imaging of the entire bale for moisture, free from interference from wire-tie artifacts.

## 4. Conclusions

The use of microwave imaging is becoming more prevalent for detection of interior hidden defects in manufactured and packaged materials. In applications for detection of hidden moisture, microwave tomography can be used to image a bale and then perform an inverse calculation to derive an estimate of the variability of the hidden interior moisture, thereby alerting personnel to damaging levels of unseen moisture before fiber degradation occurs. One impediment to this type of imaging is when the packaging utilizes metal strapping ties, as the reduced aperture of the imaging antennas, size and close proximity of the metal bale ties combine to create significant Mie scattering that causes destructive and constructive interference as the material is conveyed past the imaging antenna array.

An experimental conveyance system was utilized that allowed for precise positioning of a bale with respect the sensing imaging antennas. By utilizing sequential measurements, where the bale is indexed forward to the next position, an incremental scan of the bale was obtained with measurements at multiple frequencies ranging from 500 MHz to 2.5 GHz. The experimental results revealed the expected variation due to the hypothesized multipath interference. However the experiment also revealed an unexpected deviation in the lateral variation in the peaks of the measurements that correlated to the wire-tie spacing. The experiments also revealed that each frequency would have to have a unique lateral offset from the metal bale-ties, which reinforces the earlier premise that a fixed channel filter would prove difficult to implement for the proposed removal of the multipath interference.

This research examined the cause and effect from both theoretical aspects through the use of FDTD simulation models as well as experiments to support the theoretical conclusions. The research provides strong support to the hypothesis that the experimental variation in permittivity as measured on bales with uniform moisture profiles is due to Mie scattering off the metal bale ties. The research also developed a solution for the removal of the influence of the metal bale ties from the measurement through the utilization of UWB scanning in the time-domain or via stepped frequency domain scanning. It will also likely be possible for a frequency-modulated-continuous-wave, “FMCW” system to perform similarly when configured for ultra-wideband scanning of permittivity, rather than a narrow-band implementation. The development presented in this paper provides a method to significantly improve the accuracy by which measurements of moisture can be obtained for the use in the generation of microwave moisture images of bales tied with metal bale ties. This work is particularly relevant for low permittivity materials that have a very localized range such as is typical of cotton where the full range of expected relative permittivity ranges is typically limited to the range {1.4 to 2.5} as the spurious Mie reflections have a larger influence on the measured response.
